# Selenium, Selenoproteins, and Heart Failure: Current Knowledge and Future Perspective

**DOI:** 10.1007/s11897-021-00511-4

**Published:** 2021-04-09

**Authors:** Ali A. Al-Mubarak, Peter van der Meer, Nils Bomer

**Affiliations:** 1 Department of Cardiology, University Medical Center Groningen, University of Groningen, Groningen, The Netherlands; 2 Department of Experimental Cardiology, University Medical Center Groningen, UMCG Post-zone AB43, P.O. Box 30.001, 9700 RB Groningen, The Netherlands

**Keywords:** Selenium, Heart failure, Selenoproteins

## Abstract

**Purpose of Review:**

(Mal-)nutrition of micronutrients, like selenium, has great impact on the human heart and improper micronutrient intake was observed in 30–50% of patients with heart failure. Low selenium levels have been reported in Europe and Asia and thought to be causal for Keshan disease. Selenium is an essential micronutrient that is needed for enzymatic activity of the 25 so-called selenoproteins, which have a broad range of activities. In this review, we aim to summarize the current evidence about selenium in heart failure and to provide insights about the potential mechanisms that can be modulated by selenoproteins.

**Recent Findings:**

Suboptimal selenium levels (<100 μg/L) are prevalent in more than 70% of patients with heart failure and were associated with lower exercise capacity, lower quality of life, and worse prognosis. Small clinical trials assessing selenium supplementation in patients with HF showed improvement of clinical symptoms (NYHA class), left ventricular ejection fraction, and lipid profile, while governmental interventional programs in endemic areas have significantly decreased the incidence of Keshan disease. In addition, several selenoproteins are found impaired in suboptimal selenium conditions, potentially aggravating underlying mechanisms like oxidative stress, inflammation, and thyroid hormone insufficiency.

**Summary:**

While the current evidence is not sufficient to advocate selenium supplementation in patients with heart failure, there is a clear need for high level evidence to show whether treatment with selenium has a place in the contemporary treatment of patients with HF to improve meaningful clinical endpoints.

**Graphical abstract:**

**Figure:**
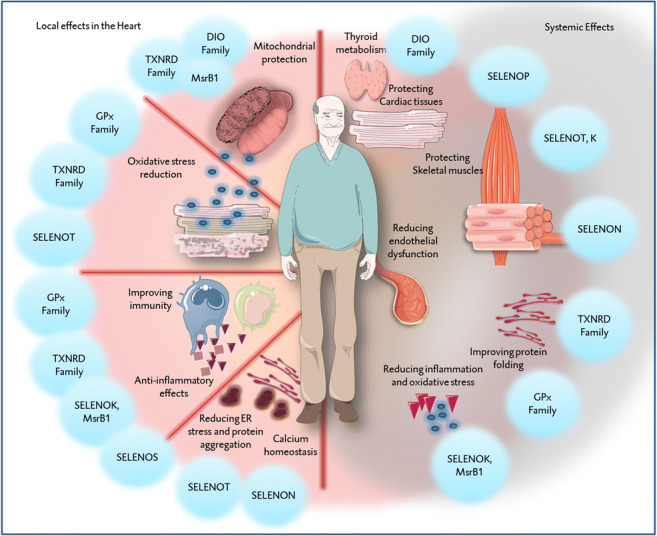
**Graphical summary summarizing the potential beneficial effects of the various selenoproteins, locally in cardiac tissues and systemically in the rest of the body.** In short, several selenoproteins contribute in protecting the integrity of the mitochondria. By doing so, they contribute indirectly to reducing the oxidative stress as well as improving the functionality of immune cells, which are in particular vulnerable to oxidative stress. Several other selenoproteins are directly involved in antioxidative pathways, next to excreting anti-inflammatory effects. Similarly, some selenoproteins are located in the endoplasmic reticulum, playing roles in protein folding. With exception of the protection of the mitochondria and the reduction of oxidative stress, other effects are not yet investigated in cardiac tissues. The systemic effects of selenoproteins might not be limited to these mechanisms, but also may include modulation of endothelial function, protection skeletal muscles, in addition to thyroid metabolism. Abbreviations: DIO, iodothyronine deiodinase; GPx, glutathione peroxidase; MsrB2, methionine-R-sulfoxide reductase B2; SELENOK, selenoprotein K; SELENON, selenoprotein N; SELENOP, selenoprotein P; SELENOS, selenoprotein S; SELENOT, selenoprotein T; TXNRD, thioredoxin reductase.

## Introduction

Heart failure (HF) is a clinical syndrome with a high morbidity and mortality, with an increasing prevalence of more than 26 million patients worldwide [1]. Despite the presence of heterogeneity within the various subtypes of HF, current treatment options mainly target the activation and consequences of the renin–angiotensin–aldosterone system (RAAS), with success in decreasing mortality and morbidity in HF with reduced ejection fraction (HFrEF), but not HF with preserved ejection fraction (HFpEF) [2]. There is evolving evidence that several pathophysiological mechanisms are involved in the development and progression of HF, including oxidative stress [3], microvascular inflammation [4], mitochondrial dysfunction [5], suboptimal metabolism, and nutrient deficiencies [6].

(Mal-)nutrition of micronutrients has great impact on human cardiomyocytes, especially on their mitochondrial function, contractility [7, 8] and their ability to recover from damage and consequently is associated with prognosis [9]. Improper micronutrient intake is frequently observed in patients with HF, affecting 30–50% of this population [10, 11]. Deficiencies in micronutrients, such as vitamin A, calcium, magnesium, selenium, iron, zinc, vitamin D, and iodine have been documented, without establishing a causative association between them and the onset of HF [6]. Of the various micronutrient deficiencies, only correcting iron deficiency by intravenous iron supplementation has found its way into the guidelines with beneficial effects on morbidity [12–15]. Notably, severe selenium deficiency in humans is associated with a rare but fatal form of dilated cardiomyopathy (DCM) that is restricted to specific geographic regions (Keshan disease) [16] that have a very low amount of selenium in the soil and therefore in food. Keshan disease is reversible with selenium supplementation [17]. Selenium is a micronutrient found in various food sources, including seafood, red meat, nuts, and grains [18]. Data from various Asian, European, and Middle Eastern countries show that dietary selenium intake as well as selenium status are suboptimal or even low in those populations [7, 19]. Selenium is essential for the enzymatic function of so-called selenoproteins. It is incorporated as the rare amino acid selenocysteine, the selenium analogue of cysteine, in which a selenium atom replaces sulfur [18]. Selenium deficiency deprives the cell and various tissues of their ability to synthesize the required amount of selenoproteins, and many health effects of low selenium intake are believed to be caused by the shortage or lack of one or more specific selenoproteins [18]. Low selenium concentrations and iron deficiency share to a large extent similar clinical predictors such as inflammation, chronic kidney disease, and lower serum albumin levels [20]. However, the association of selenium on prognosis might be even more pronounced as only low selenium levels are associated with higher all-cause mortality when both selenium and iron levels were taken into consideration [20].

In this review, we aim to summarize the current knowledge about selenium deficiency and selenoproteins in the context of HF. Furthermore, we will elaborate shortly on the future perspective of selenium supplementation trials in patients with HF.

### Selenium Deficiency and HF: Current Clinical Knowledge

Previous studies suggested that severe selenium deficiency in humans is associated with cardiomyopathy (Keshan disease) [17]. This disease has similar clinical characteristics as idiopathic DCM, but with strong geographic distribution [21]. Treatment with selenium mitigated the clinical manifestations in patients with the disease and the government implemented nutritional policies promoting oral selenium supplementation, which virtually eliminated Keshan disease in areas where it was endemic [21–23].

### The definition of “selenium deficiency and suboptimal selenium status”

There is no consensus about a specific cut-off to define selenium deficiency and what is considered a normal range is usually defined based on selenium levels in healthy population in specific geographical area [24, 25]. In these populations, serum selenium concentrations <70 μg/L are considered to indicate deficiency [26, 27]. Circulating selenoproteins have been established as protein biomarkers of selenium status in subjects with marginal selenium status, i.e., glutathione peroxidases and selenoprotein P (SELENOP), as they correlate almost linearly with selenium intake [26]. However, with a sufficiently high selenium supply, these protein biomarkers reach plateau levels at serum selenium concentrations of approximately 90 to 125 μg/L [28, 29]. Supporting this, selenium concentrations below 100 μg/L are associated with a poorer quality of life and exercise capacity, and an impaired prognosis in patients with worsening HF [7••]. In addition, in a meta-analysis that included 16 observational studies, an inverse association between cardiovascular events and selenium levels up to 106 μg/L was reported [30, 31]. Taken together, these findings suggest that optimal selenium might be higher than previously thought and that patients with selenium levels <100 μg/L should be considered to a certain extent selenium deficient, or at least suboptimal.

### Observational Studies on Selenium Status

Measuring selenium concentrations routinely is laborious and costly, and therefore the available data on selenium measurements in patients with HF is scarce. Only very recently, serum selenium concentrations were measured in a large European cohort of patients with worsening HF (*N* = 2328). Approximately 70% of patients with HF showed selenium serum levels <100 μg/L [7, 20]. These patients were found to have a poorer quality of life, poor exercise capacity, and a worse prognosis [7••]. Older age, lower levels of albumin, worse kidney function, higher levels of NT-proBNP, and the presence of orthopnea and iron deficiency were established as independent predictors of selenium levels <100 μg/L in patients with worsening HF [20]. These observations are supported by similar findings in other small cohorts [32–35]. While these studies [7, 32–35] focused mainly on HF with reduced ejection fraction (HFrEF), one small cohort-study aimed to compare selenium levels between HFrEF and HF with preserved ejection fraction (HFpEF) [36]. This study [36] showed that HFrEF patients have significantly lower selenium levels (HFrEF, 73.6 ± 7.5 μg/L) compared to those with HFpEF (77.7 ± 4.9 μg/L), but only selenium levels <90μg/L were reported in this study [36].

Several meta-analyses have assessed the pooled association between selenium and heart disease. Firstly, Flores-Mateo et al. showed that higher selenium concentrations were associated with a pooled relative risk (RR) of 0.85 (95% CI 0.74, 0.99) for acute coronary artery disease in prospective observational studies and a pooled RR of 0.43 (95% CI 0.29, 0.66) in case–control studies [37]. This conclusion was confirmed in a more recent analysis performed by Zhang et al., where patients with highest category of selenium status (median 101.5 μg/L) had a RR of 0.87 (95% CI 0.76, 0.99) for the development of cardiovascular disease (CVD) compared to the lowest category (median selenium 53.7 μg/L) with more pronounced inverse associations in studies with selenium levels <106 μg/L [30]. In addition, in a subsequent meta-analysis that analyzed cardiovascular mortality and incidence separately, Kuria et al. concluded that, in five cohort studies, high selenium status significantly reduced cardiovascular mortality (RR 0.75; 95% CI 0.64, 0.87) and incidence (RR 0.80; 95% CI 0.70, 0.92) compared to low selenium status [38••]. While these studies mainly had a retrospective design, evidence from prospective studies in healthy subjects that develop HF in a later stage is still lacking.

### Selenium Supplementation Trials

Only two, small, randomized controlled trials (RCTs) aimed specifically at patients with HF have been performed in Iran. Garakyaraghi et al. supplemented 32 HFrEF patients with combined 90 mg CoQ10 and 200 μg selenium per day for 3 months. This led to significant improvement of NYHA class, left ventricular ejection fraction, and myocardial performance index compared to the control group [39]. Besides the low number of included subjects, it should be acknowledged that patients were sub-optimally treated for HF as they were mainly receiving ACE inhibitors or beta-blockers, while around 50% were using diuretics. In addition, it cannot be concluded whether these effects are a result of selenium supplementation or CoQ10 use as they were given coincidentally, especially as no selenium levels have been measured. Another study with comparable patient inclusion criteria supplemented 200 μg selenium per day alone for 12 weeks. Mainly lipid profiles have been evaluated in this study [40], which showed significant reduction of LDL-cholesterol, insulin levels, and C-reactive protein, and on the other hand increased HDL levels, total antioxidant capacity, and glutathione concentrations [40]. Of particular interest, a recent meta-analysis showed significant reduction of Keshan disease in endemic areas in the groups that received selenium supplementation [22•], indicating the potential causative role of selenium deficiency in inducing HF in these patients. Given that more than 70% of patients with Keshan disease get the diagnosis before the age of 55 [41] and had average blood selenium levels of around 25 μg/L [42], it may be hypothesized that the consequences of low selenium levels might appear in older age in populations with higher levels as seen in Europe [7, 20].

In addition, the KISEL-10 study is a RCT that assessed selenium and CoQ10 supplementation for a period of 48 months in healthy individuals in a country known to have low selenium levels (Sweden) [43]. This study reported a significant reduction of cardiovascular mortality, but not all-cause mortality, in individuals aged between 70 and 88 years. However, it should be noted that of the 443 randomized patients in this previous study, only 228 patients completed the study. Of note, withdrawals were balanced in both groups and the reasons for drop-out did not differ significantly [43]. In addition, in a post hoc analysis of the same cohort, no significant reduction in mortality rate was observed in patients with selenium levels >85 μg/L, which might be attributed to the low number of patients with such selenium status (*N =* 47) [44]. Although the targeted population in this study were healthy elderly individuals, the authors reported significantly lower NT-proBNP levels in the interventional group which remained at approximately the same reduced level after 48 months [43]. Echocardiographically, there were no significant differences between the groups. This might be attributed to some extent to the health status of the dropped-out individuals since there were lower ejection fractions in the active group compared to the placebo group at the inclusion of the study, as reported by the investigator [43].

On the other hand, findings from several RCTs are inconclusive. A Cochrane review concluded that selenium supplementation should not be used in primary prevention of CVD as there was no significant effects on all-cause mortality (RR 0.97; 95% CI 0.88, 1.08), CVD mortality (RR 0.97; 95% CI 0.79, 1.2), or all CVD events (RR 1.03; 95% CI 0.95, 1.11) [45]. These findings are in line with previous meta-analyses that examined RCTs [30, 37, 38], with the exception of a recent meta-analysis where the authors examined the effect of antioxidant mixtures in relation to mortality, using 43 studies in the final analysis (*N=* 114,146). Antioxidant mixtures were defined as a combination of two or more of the following molecules: vitamin A, retinol, β-carotene, vitamin C, vitamin E, selenium, zinc, and copper [46]. They concluded that there was significant reduction of cardiovascular mortality and all-cause mortality when selenium is included in the antioxidant mixture (RR 0.77; 95% CI 0.62, 0.97 and RR 0.90; 95% CI 0.82, 0.98, respectively), findings that were not observed when selenium was not part of the antioxidant mixture. An important factor that may influence the aforementioned analyses is the location where the included studies were conducted. The majority of patients (>90%) were from selenium-replete populations (Northern America) that most likely have optimal functioning of their selenoproteins since intakes are high in Canada, the USA, and Japan (>100 μg/day), and much lower in some parts of Europe (~40 μg/day) [31]. As such, these patients may show less benefit of selenium supplementation. Supporting this, Kuria et al., in a sub-analysis, reported a significant reduction of cardiovascular mortality in studies conducted in Asia (RR 0.59; 95% CI 0.45, 0.79) and Europe (RR 0.55; 95% CI 0.45, 0.68), but not in the USA (RR 0.93; 95% CI 0.82, 1.05) [38••].

### The U-Shape Observation

Several studies suggested the presence of a U-shape in terms of the association between selenium levels and mortality, with increasing CVD risk in patients with selenium levels >145–150 μg/L [30, 31, 45]. This notion might be yet debatable. The evidence seems to be insufficient to examine the relationship when selenium levels exceed 150 μg/L [30]. For example, in a Danish multiple-dose RCT in a relatively healthy population, patients who received 200 μg/day for 5 years had lower mortality compared to the placebo group (*N* = 119–126 patients in each group). The baseline selenium of this group was 87.5 μg/L, which increased to 225 μg/L after 5 years. On the other hand, the group who received daily 300 μg selenium had selenium plasma levels of 284 μg/L and significantly higher mortality compared to the placebo group [47], supporting the notion of U-shape but with much higher selenium levels than previously suggested. Further research is needed for a better understanding of this concept as it might present oversimplification [48]. Several genetic and environmental factors might play a role in the way the human body tolerates low or high selenium levels, especially as not all people with (very) high selenium have symptoms of selenium toxicity [48]. These findings suggest that there is “space to win” with supplementation since most patients with HF have selenium levels <100 μg/L. However, cautiousness should be taken into account.

### Selenium and Selenoproteins: Comorbidities in HF Pathology

It remains to be clarified whether selenium deficiency in patients with HF is just a marker of worse disease severity, or causative for the development and progression the HF. Furthermore, the pathophysiological and molecular mechanisms affected by selenium deficiency are underrepresented in current HF research, and demands more attention. Selenium is co-translationally incorporated into the polypeptide chain as component of the amino acid selenocysteine (Sec), the 21st amino acid in the genetic code, which is encoded by TGA [49]. Proteins including Sec in their polypeptide chain are defined as selenoproteins [50, 51]. In the human genome, there are 25 so-called selenoproteins. Selenoproteins are relatively highly conserved as 24 of them are expressed in mice, although there might be some differences in their functional importance between human and mice, as suggested by a recent study [52]. Sufficient selenium levels are essential in order to synthesize the required amount of selenoproteins, especially as the lack or shortage of these proteins may have consequences in the context of the heart [18]. These selenoproteins have important functions in antioxidant defense, thyroid metabolism, protein folding and immunity (Table 1). Only few selenoproteins have been studied in clinical settings in relation to HF or CV diseases.

**Table 1:** List of selenoproteins and selenium-incorporating proteins with their functions

Name of protein	**Abbreviation**	**Functions**
Glutathione peroxidase 1	GPx1, GPX1	Considered the most important cellular redox regulator [18]
Glutathione peroxidase 2	GPx2, GPX2	Regulates redox homeostasis, essential for peroxide reduction in the gut, involved in tissue regeneration and cell proliferation [18]
Glutathione peroxidase 3	GPx3, GPX3	Regulates redox homeostasis, essential for peroxide reduction in the blood [18]
Glutathione peroxidase 4	GPx4, GPX4	Regulates redox homeostasis, important for phospholipid peroxide reduction [18, 53, 54]
Glutathione peroxidase 6	GPx6, GPX6	Involved redox homeostasis mainly in olfactory epithelium [18, 53, 54]
Iodothyronine deodinase 1	DIO1	Converts T4 to T3, primarily in the thyroid, liver and kidney and regulates the circulating levels of thyroid hormone [18, 53, 54]
Iodothyronine deodinase 2	DIO2	Converts T4 to T3, it is tissue specific (it is abundantly found in the thyroid gland and in the heart) [18, 53, 54]
Iodothyronine deodinase 3	DIO3	Inactivates T4 and T3 [18, 53, 54]
Thioredoxin reductase Type I	TXNRD1, also named: TR1, TrxR1	Reduces oxidized thioredoxin, it functions mainly in the cytosol [18, 53, 54]
Thioredoxin reductase Type II	TRXRD2, also named: TR3	Reduces oxidized thioredoxin, it functions mainly in the mitochondria [18, 53, 54]
Thioredoxin reductase Type III	TRXRD3, also named: TR2,TGR	Reduces oxidized thioredoxin, it is found mainly in the testis [18, 53, 54]
Methionine-R-sulfoxide reductase	MsrB1, SelR, SelX, MSRB1	Reduces oxidized methionine residues on proteins [53, 54], may be involved in inflammation [55]
Selenoprotein F	SELENOF, also named: Sep15	Regulation of redox homeostasis in the ER, may be involved in protein folding regulation [54]
Selenoprotein H	SELENOH, also named, SelH	Not yet fully established functions [54], involved in redox regulation and as a transcription factor [53]
Selenoprotein I	SELENOI, also named Sell, SEPI, EPT1	Not yet fully established functions, may involve in phospholipid biosynthesis [53] [54],
Selenoprotein K	SELENOK, also named: Selk	Involved in immune cell processes, cellular calcium hemostasis as well as mechanisms related to ER stress [53]
Selenoprotein M	SELENOM, also named: SelM, SEPM	Not yet established functions, may be involved in neuroprotection and calcium hemostasis [54], may have roles in body weight and energy metabolism [53]
Selenoprotein N	SELENON, also named: SelN, SepN, SEPN1	Has roles during muscle development [53], involved in calcium hemostasis [56]
Selenoprotein O	SELENOO, also named: SelO	Not yet established functions [54], may have function in redox regulation [53]
Selenoprotein P	SELENOP, also named: SelP, SEPP1	Mainly transports selenium to tissues [18, 53, 54]. May have anti-fibrotic effects [57]
Selenoprotein S	SELENOS, also named: SelS, SEPS1, VIMP	May involve in mechanisms related to ER stress and protection against protein aggregation [53]
Selenoprotein T	SELENOT, also named: SelT	Not yet fully established functions, may have influence on cell structure organization and cell adhesion characteristics [54], involved in calcium mobilization in the ER [53] and in protein folding regulation [58, 59]
Selenoprotein V	SELENOV, also named: SelV	Not yet fully established functions [54], expressed mainly in the testes [53]
Selenoprotein W	SELENOW, also named: SelW, SEPW1	Not yet fully established functions [54], may have roles in antioxidant defense and muscle growth [53]. May stimulate cell cycle progression by facilitating G1/S transition [60]

### Transport and Surrogate Marker of Selenium Status

#### Selenoprotein P

Among the well-studied selenoproteins is selenoproten P, the main carrier of selenium in the blood. Recently, in the context of acute HF, it has been reported that patients with lower SELENOP levels had greater risk for 30-day rehospitalization (HR 4.29; 95% CI 1.59, 11.6), 1-year mortality (HR 4.13; 95% CI 1.64, 10.4), and a composite endpoint of death or mortality within 30 days (HR 4.80; 95% CI 1.80, 12.8) compared to patients with higher levels [61]. Similarly, in a large Swedish prospective cohort study in primary preventive setting, patients with high SELENOP levels (>5.9 mg/L) had significantly lower risk for all-cause mortality (0.57, 0.48–0.69), cardiovascular mortality (0.52, 0.37–0.72), and first cardiovascular event (0.56, 0.44–0.71) [62]. SELENOP might be a good surrogate marker for blood selenium measurements, circumventing the costly and labor-intensive method of inductively coupled plasma-mass spectrometry (ICP-MS) that is normally used for diagnostics. As SELENOP increases gradually until serum selenium concentration reaches 125 μg/L upon selenium supplementation [31], these data emphasize the importance of sufficient selenium status.

### Oxidative stress and Mitochondrial Dysfunction

#### Glutathione Peroxidases (GPx)

Glutathione peroxidase (GPx) enzymes are essential selenoproteins involved in the antioxidant defense and have been extensively investigated in pre-clinical models, which have been reviewed elsewhere [63]. GPx enzyme activity was lower in patients with HF as compared to healthy controls when evaluating different compartments within the heart [64] as well as in their skeletal muscles [65]. Moreover, reports that are based on blood samples indicate low GPx activity and levels in patients with HF [66–68], while higher GPx levels lead to better survival as observed in a small study in patients who received left ventricular assist device (LVAD) [69]. Of note, several studies did not show a rise in GPx levels even after initiating or optimizing HF treatments [70–72]. Since HF is recognized by increased oxidative stress [3], this addresses a new therapeutic opportunity, especially in patients with low selenium levels because it was reported that selenium supplementation can increase GPx levels in cardiac tissues [33]. As mentioned above, in order to reach the plateau level of GPx levels, a minimum of selenium level >125 μg/L is required, emphasizing the potential benefits of selenium supplementation in the context of HF.

#### Thioredoxin Reductases (TXNRD1, TXNRD2, and TXNRD3)

TXNRD1 and TXNRD2 are available in the cytosol and the mitochondria of mammalian cells, are essential for cellular redox balance, and are both highly expressed in the heart. Despite that TXNRD protein levels in the blood of patients with HF did not differ significantly from healthy controls [73], TXNRD1 activity in cardiac tissues in patients with ischemic heart disease is significantly reduced [74]. This may indicate increased need for this protein in the cardiomyocytes in stressed context. Moreover, mutations TXNRD2 have been reported to cause DCM [75, 76], while specific single-nucleotide polymorphisms (SNPs) can predispose patients to a higher risk of myocardial infarction [77].

### Thyroid hormone synthesis and metabolism

#### lodothyronine Deiodinases (DIO1, DIO2, and DIO3)

Iodothyronine deiodinases regulate thyroid hormone levels at plasma level (DIO1) as well as at tissue and cellular level, converting thyroxine, T_4_, to the active form, 3,3′,5-triiodo l-thyronine, T_3_ (DIO2), and vice versa (DIO3). Data about these selenoproteins are limited in the context of HF. T_3_ is a powerful regulator of cardiac contractility through its effect on myosin isoforms and calcium handling proteins [78]. Furthermore, the T_3_ hormone stimulates cardiac mitochondrial biogenesis increasing myocardial mitochondrial mass, mitochondrial respiration, oxidative phosphorylation (OXPHOS), enzyme activities, mitochondrial protein synthesis (stimulated through T_3_), cytochrome, phospholipid, and mtDNA content [79]. “Low T_3_ syndrome,” defined as low levels of T3 with normal levels of TSH and FT4, is a condition that is associated with increased composite end point of ventricular assist device placement, heart transplantation, or death in patients with pre-existing HF (HR 2.12; 95% CI 1.65, 2.72; *P* < 0.001) [80] and the lack of optimal activity of deiodinases may play a role in this syndrome [81].

### Protein Folding and ER Stress

Accumulation of misfolded proteins in the endoplasmatic reticulum (ER) contributes to vascular and cardiac diseases through the ER stress response, a protein quality control system that resides in the ER [82]. SELENOT is a selenoprotein that resides in the ER and is expressed accordingly in the heart [83]. As an essential ER protein, SELENOT is required for adaptation in stressful conditions like unfolded protein response [58, 59]. It was shown to have protective effects in an ischemia–reperfusion model [84] and may suppress oxidative stress and apoptosis [85]. Since ER stress and proteotoxicity are involved in the molecular pathology of cardiac dysfunction [86], reducing it may be favorable. SELENON is a long transmembrane selenoprotein that is also localized in the ER of the heart [87]. It can sense the luminal calcium level and thereby modulate the SERCA-mediated replenishment of ER calcium stores [56]. SELENON deficiency is associated with various congenital myopathies, and these patients have impaired glucose metabolism with defective insulin-dependent glucose uptake, which in turn may lead to insulin resistance [88, 89].

### Inflammation and Immunity

Selenium is essential for the efficient and effective operation of many aspects of the immune system in humans [90]. Mitochondrial matrix protein methionine sulfoxide reductase (MsrB2) is a selenoprotein that is readily regulated by dietary selenium. It was shown that MsrB2 has anti-inflammatory effects and is required for the maximal induction of two anti-inflammatory cytokines, IL-10 and IL-1RA [55]. Furthermore, it may play an essential role in mitophagy, the removal process of damaged mitochondria [91]. Next to this, SELENOK is suggested to facilitate the differentiation of T cells and the functionality of other immune cells [92, 93], which is of particular importance as patients with HF show higher incidence of infections and sepsis [94].

Inflammation and immunity are related to endothelial dysfunction that can be observed in HF [95]. Most studies on the role of selenium in endothelial processes show selenium-dependent endothelial functions and explain how cells and tissues adapt to inflammatory insults [96]. Several experimental studies showed that selenium reduces endothelial dysfunction and has a cytoprotective role [96], through SELENOS [97, 98] and SELENOP [99]. Moreover, a recent sub-study from KiSel-10 study showed that selenium and CoQ10 significantly reduced the levels of von Willebrand factor (vWF) and plasminogen activator inhibitor-1 (PAI-1), both of which are seen increased in diseases with vascular dysfunction [100]. Lastly, several reports indicated that immune cells are particularly vulnerable to the imbalance between antioxidants and free radicals (i.e., oxidative stress), leading to their malfunction [101, 102]. It is established that selenium concentrations within the immune cells themselves have influence on the half-life of reactive oxygen species (ROS) as many selenoproteins neutralize ROS via various pathways [103]. Selenium has the potential not only to promote differentiation of T-cell subtypes in HF but also to improve their functionality by reducing the intracellular oxidative stress.

There is still much unknown of the effect of the other selenoproteins (selenoproteins F, H, I, M, O, V, W) [49] and their mechanisms of action are still to be further investigated. It will be of interest to examine their expressions in cardiac tissues of patients with HF as there are no specific reports in that regard. This may provide hints for potential therapeutic windows since the epidemiological evidence regarding Keshan disease supports the preventive effects of selenium supplementation and these proteins may modulate yet to be targeted pathophysiological processes in HF.

## Conclusion and Future Perspective

Suboptimal selenium levels are common among patients with HF, also in the developed world [7, 20]. Patients with low selenium levels would have most likely low expression of selenoproteins, affecting the availability and the functionality of these proteins. Loss or sub-optimal function of specific selenoproteins might have detrimental effects as reported in (pre)clinical studies. Nevertheless, there are yet no specific cohort studies that examined low selenium levels and incident of HF prospectively. Selenoproteins may exert their beneficial effects not only locally in cardiac tissues but also systematically. They may do so through several mechanisms that are in fact disrupted in HF, including maintaining healthy mitochondria, redox reduction, anti-inflammatory effects, improving immune cells functionality, improve thyroid metabolism, and reducing ER stress. Since HF is a systematic disease, these effects suggest that selenium supplementation may have additional systematic improvements. The current evidence is not (yet) strong enough to encourage selenium supplementation in clinical practice. It is, however, encouraging enough to start large well-designed randomized clinical trials. The focus in such future studies should be on patients with selenium levels <100 μg/L as they may benefit the most from the supplementation. In addition, whether IV or oral supplementation is better in HF, is a matter that needs investigation, especially when taking the similarities with iron into consideration [20], as oral supplementation of iron is not effective in HF [12•].

If proven to be effective, selenium supplementation may lead to a potentially inexpensive and cost-effective supplementation strategy that can be applied in a personalized manner, leading to less clinical symptoms and improved prognosis.
